# PAM50 intrinsic subtypes, risk of recurrence score and breast cancer survival in HIV-positive and HIV-negative patients—a South African cohort study

**DOI:** 10.1007/s10549-023-06969-1

**Published:** 2023-06-02

**Authors:** Boitumelo Phakathi, Therese Dix-Peek, Eunice Van Den Berg, Caroline Dickens, Sarah Nietz, Herbert Cubasch, Maureen Joffe, Alfred I. Neugut, Judith S. Jacobson, Paul Ruff, Raquel Duarte

**Affiliations:** 1 Department of Surgery, Nelson R Mandela School of Medicine, University of Kwa-Zulu Natal, 719 Umbilo Road, Durban, 4001 South Africa; 2 Department of Internal Medicine, University of Witwatersrand Faculty of Health Sciences, 7 York Road, Parktown, Johannesburg, South Africa; 3 Department of Anatomical Pathology, University of Witwatersrand Faculty of Health Sciences, 7 York Road, Parktown, Johannesburg, South Africa; 4 Department of Surgery, University of the Witwatersrand Faculty of Health Sciences, 7 York Road, Parktown, Johannesburg, 2193 South Africa; 5 Strengthening Oncology Services Research Unit, University of the Witwatersrand Faculty of Health Sciences, Johannesburg, South Africa; 6 Batho Pele Breast Unit, Chris Hani Baragwanath Academic Hospital, 26 Chris Hani Road, Diepkloof, Soweto, 1860 South Africa; 7 WITS/SAMRC Common Epithelial Cancers Research Centre (CECRC, Cape Town, South Africa; 8 Herbert Irving Comprehensive Cancer Center, Vagelos College of Physicians and Surgeons, Columbia University, New York, USA; 9 Department of Epidemiology, Mailman School of Public Health, Columbia University, New York, USA; 10 Division of Medical Oncology, University of the Witwatersrand Faculty of Health Sciences, 7 York Road, Parktown, Johannesburg, South Africa

**Keywords:** Breast cancer, HIV, Gene expression assay, Intrinsic subtypes, Risk of Recurrence score, Survival

## Abstract

**Purpose:**

Treatment decision making for patients with breast cancer increasingly depends on analysis of markers or systems for estimating risk of breast cancer recurrence. Breast cancer intrinsic subtypes and risk of recurrence (ROR) scores have been found to be valuable in predicting survival and determining optimal treatment for individual patients. We studied the association of breast cancer survival with the PAM50 gene expression assay in HIV-positive and HIV-negative patients.

**Method:**

RNA was extracted from formalin-fixed paraffin-embedded specimens of histologically confirmed invasive carcinoma and was purified using the AllPrep® DNA/RNA FFPE kit, Qiagen (Hilden, Germany). The NanoString RUO PAM50 algorithm was used to determine the molecular subtype and the risk of recurrence score of each sample. The overall and disease-free survival were determined with comparison made among HIV-positive and -negative patients. We then generated Kaplan–Meier survival curves, calculated p-values and estimated hazard ratios and their 95% confidence intervals using Cox regression models.

**Results:**

Of the 384 RNA samples analysed, 98.4% met the required RNA quality standard and the specified QC threshold for the test. Luminal B was the most common PAM50 intrinsic subtype and 82.1% of patients were at high risk for disease recurrence based on ROR score. HIV infection, PAM50-based HER2-enriched and basal-like intrinsic subtypes, and high ROR were associated with poor overall and disease-free survival. HIV-positive patients with luminal A & B subtypes had significantly worse survival outcomes than HIV-negative luminal patents.

**Conclusion:**

Aggressive tumour biology was common in our cohort. HIV infection, PAM50 HER2-enriched,basal-like intrinsic subtypes and high ROR score were associated with poor overall and disease-free survival. HIV infection impacted survival in patients with luminal subtypes only.

## Introduction

In recent years, breast cancer has surpassed lung cancer in mortality and has become the most frequently occurring malignancy, accounting for 24.5% of all malignancies, among women globally [1]. In 2020, it accounted for more than 680 000 reported deaths globally, more than half of them in low- or middle-income countries (LMICs) [1]. In high-income countries, breast cancer survival is reported to be more than 90%, but in sub-Saharan African countries, survival has been reported to be about 50% (48%–53%) [2]. In a recently published urban-based, South African study, the 4-year breast cancer overall and disease-free survival proportions were reported to be 53.5% and 55.8%, respectively [3].

HIV infection has been associated with increased mortality among patients diagnosed with cancer [4–7]. Phakathi et al. reported that the overall and disease-free survival for breast cancer was worse among HIV-positive patients [3]. Moreover, among the HIV-positive patients, survival was better among those who were on anti-retroviral therapy (ART) at the time of breast cancer diagnosis [3]. Whether HIV infection directly affects breast cancer progression is not yet known. However, younger age, more advanced disease at breast cancer diagnosis, and greater difficulty in completing systemic therapy observed among HIV-positive patients, may contribute to their poorer outcomes [4–8].

Treatment decision making for breast cancer patients depends on their estimated risk for disease recurrence. Traditionally, such estimates have been based on clinical-pathological factors. But in the past few decades, several predictive tools or systems have been developed to improve the accuracy and usefulness of such estimates [9]. For example, immunohistochemistry looks at specific proteins expressed on tumour cells; in-situ hybridisation assesses gene amplification; and reverse transcription-polymerase chain reactions examine gene transcription [10, 11]. Immunohistochemistry and the PAM50 gene expression assay identify intrinsic subtypes of breast cancer.

The prediction analysis microarray (PAM50) gene expression assay measures mRNA expression of 50 cancer-related genes; the assay classifies the tumour by breast cancer intrinsic subtype and generates its risk of recurrence (ROR) score [12]. The ability of PAM50 scoring to prognosticate and predict recurrence and metastasis exceeds that of scoring based on the traditional clinico-pathological characteristics of breast cancer [12]. Moreover, the ROR score has been reported to add more prognostic information than the clinical treatment score, recurrence score (Oncotype Dx) and IHC-4 in both node-negative and node-positive, HER-2 negative early breast cancer [9, 12]. It has also achieved analytical validation and level 1 clinical validation and has shown clinical utility and effectiveness in predicting the risk of recurrence in post-menopausal women [12–15]. In a study in Canada, among patients in the high-risk group, PAM50 was able to distinguish those who would respond well to chemotherapy from those who would not by intrinsic subtype [16]. Similarly, in a Norwegian study, the PAM50 assay identified low-risk patients who could be followed safely by observation and would not derive an additional survival benefit from adjuvant hormonal therapy. This group of patients had a breast-cancer specific survival of 96.3% after 15 years of follow-up [12]. The PAM50 assay also identified some patients in the intermediate risk group who could derive the same survival benefit from adjuvant hormonal therapy as the low-risk group [12]. In a US sample, the PAM50 assay predicted the effectiveness of adjuvant chemotherapy as well as that of neoadjuvant chemotherapy; with an estimated negative predictive value for a complete pathological response of 97% [17]. Moreover, the PAM50 ROR was able to predict which patients with early stage breast cancer,, ER positive/ HER-2 negative, node-positive breast cancer could be safely treated with adjuvant hormonal therapy only as well as those who could benefit from chemotherapy [18]. The assay was also found to be cost-effective when compared to current clinical practice and other molecular assays [19].

However, as far as we can determine, our study is the first to report on breast cancer survival by PAM50 intrinsic subtype & ROR in HIV-negative and HIV -positive patients of South Africa. We hypothesised that HIV-positive women with breast cancer would have a more aggressive tumour phenotype than HIV-negative patients, and therefore poorer survival.

## Methodology

Among participants in the South African Breast Cancer and HIV Outcomes (SABCHO) cohort study, we selected formalin-fixed, paraffin-embedded (FFPE) specimens of histologically confirmed invasive carcinoma from age-matched, HIV-positive and -negative patients. We obtained mastectomy/wide local excision specimens from patients who had primary surgery, and core biopsy specimens from patients who had primary chemotherapy. We retrieved the FFPE breast tissue blocks from the archives of the National Health Laboratory Service (NHLS). The pathologist examined a hematoxylin and eosin (H&E) stained slide, marked the area of invasive breast cancer suitable for the test, and sent the slides to the Molecular Laboratory, at the University of Witwatersrand, where the molecular work was undertaken.

We extracted and purified the RNA successfully using the AllPrep® DNA/RNA FFPE kit, Qiagen (Hilden, Germany). We measured the extracted RNA on the Nanostring nCounter Analysis System (Nanostring Technologies, Seattle, WA) and processed the samples using the NanoString nCounter Prep Station and digital analyser. PAM50 analysis was done on 384 samples, 6 (1.65%) failed the QC for the PAM50 assay and one sample was excluded because of a lack of clinical data, thus the total study population included in this analysis was 377. Of these 377, one patient had an unknown HIV status and four were excluded from the survival analyses as they lacked a follow-up period. We used NanoString RUO PAM50 algorithm to determine the molecular subtype and the ROR of each sample. We obtained the data on demographic characteristics, clinical stage at presentation, PAM50 intrinsic subtypes (luminal A, luminal B, HER2-enriched, and basal-like), ROR, HIV status, CD4 count, viral load, and ART use from the electronic breast cancer database. We categorised each patient based on her ROR score as [12]:

- Low risk: ROR ≤ 40
- Intermediate risk: ROR 41 – 60, pN0
- High risk: ROR 41 – 60, pN1 or ROR > 60

We defined overall survival as the interval from the date of breast cancer diagnosis to the date of death from any cause, and disease-free survival as the interval from the date of breast cancer diagnosis to the date of radiologically & histologically confirmed disease recurrence or death from any cause [3]. Patients with metastatic breast cancer at the time of diagnosis were not included from the analysis of the disease-free survival [3]. The date of death was documented as indicated in the medical records or provided by the family member, for patients who have died. Associations between the clinical and demographic characteristics in HIV-positive and HIV-negative participants were evaluated using a chi-squared test. Kaplan–Meier survival curves were generated and p-values were calculated using a log-rank test of equality. Both adjusted and unadjusted (crude) HRs as well as their 95% confidence intervals were estimated using Cox regression models. STATA v14.2 and the stset suite of commands were used for the data analysis, with *p* < 0.05 considered to be statistically significant [3]. The study’s ethics approval was obtained from the Human Research Ethics Committee (Medical) at the University of Witwatersrand (clearance numbers: M161130 and M150351).

## Results

A total of 377 patients (176 HIV-positive and 200 HIV-negative patients; 1 HIV status unknown) were included in the final analysis. (Table 1). The median age of the cohort was 48 years, and the HIV-positive patients were younger than the HIV-negative patients. A total of 213 (56.5%) patients had advanced disease at the time of diagnosis and 81.7% of patients had a high risk for disease recurrence. Luminal B was the most common intrinsic subtype in overall and among the HIV-negative patients.

**Table 1:** Demographic and clinical characteristics and PAM 50 intrinsic subtypes by HIV status

	Overall (n = 377)	HIV-positive^*^ (n = 176)	HIV-negative^*^ (n = 200)	p-value
Age at diagnosis (years)	48 (42–57)	46 (41–53)	51 (44–59)	** < 0.001**
Stage at diagnosis^†^				p = 0.404
Stage 1	13 (3.5%)	7 (4.0%)	6 (3.0%)	p = 0.605
Stage 2	151 (40.1%)	65 (36.9%)	86 (43.0%)	p = 0.231
Stage 3	171 (45.4%)	80 (45.5%)	90 (45.0%)	p = 0.930
Stage 4	42 (11.1%)	24 (13.6%)	18 (9.0%)	p = 0.154
Early Stage (1 and 2)	164 (43.5%)	72 (40.9%)	92 (46.0%)	p = 0.321
Advanced Stage (3 and 4)	213 (56.5%)	104 (59.1%)	108 (54.0%)	
Molecular Subtype				p = 0.115
Luminal A	73 (19.4%)	39 (22.2%)	34 (17.0%)	p = 0.207
Luminal B	122 (32.1%)	47 (26.7%)	75 (37.5%)	**p = 0.026**
HER2-enriched	89 (23.9%)	41 (23.3%)	47 (23.5%)	p = 0.963
Basal-like	93 (24.7%)	49 (27.8%)	44 (22.0%)	p = 0.190
Risk of recurrence				p = 0.677
Low	33 (8.8%)	14 (8.0%)	19 (9.85%)	p = 0.597
Intermediate	36 (9.6%)	15 (8.5%)	21 (10.5%)	p = 0.516
High	308 (81.7%)	147 (83.5%)	160 (80.0%)	p = 0.379
ROR score (median (IQR))	67 (55–80)	65.5 (53–79)	68 (56–82)	p = 0.222
Lost to Follow-up	55 (14.6%)	21 (11.9%)	33 (16.6%)	p = 0.200
For HIV-positive patients				
Detectable viral load		66 (43.1%)		
Viral load, copies/ml (median (IQR))		2195 (215–59,096)		
CD4 count, cells/mm^3^ (median (IQR))		450.5 (271–677.5)		
Duration of HIV sero-positivity				
≤ 1 year		66 (38.2%)		
> 1 year		107 (61.9%)		
On ART		124 (71.3%)		
Duration of ART use				
≤ 1 year		20 (17.0%)		
> 1 year		98 (83.1%)		

*1 (0.3%) patients had an unknown HIV status

^†^Staging was performed clinically during the initial diagnostic exam

### Overall survival

**Fig. 1:**
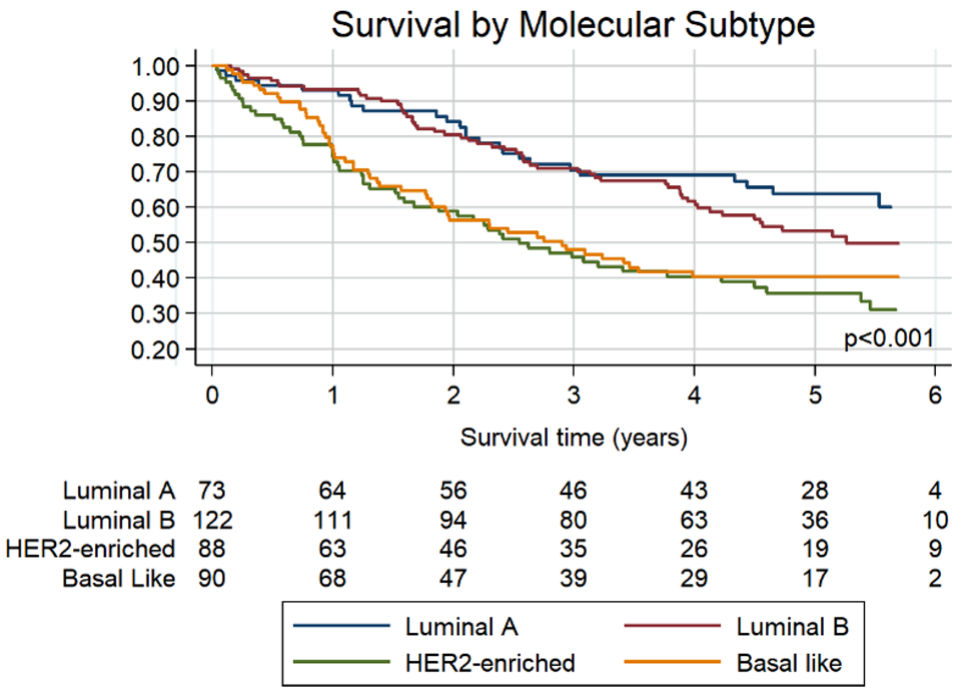
Overall survival by molecular subtype for 373 black South African patients subtyped using the PAM50 assay. The figure shows the number at risk by subtype for each time point. Unadjusted hazard ratios (95% confidence intervals (CIs)) compared to Luminal A: Luminal B 1.24 (0.78 – 1.98), *p* = 0.365; HER2-enriched 2.38 (1.49 – 3.80), *p* < 0.001; Basal-like 2.07 (1.29 – 3.33), *p* = 0.003

**Fig. 2:**
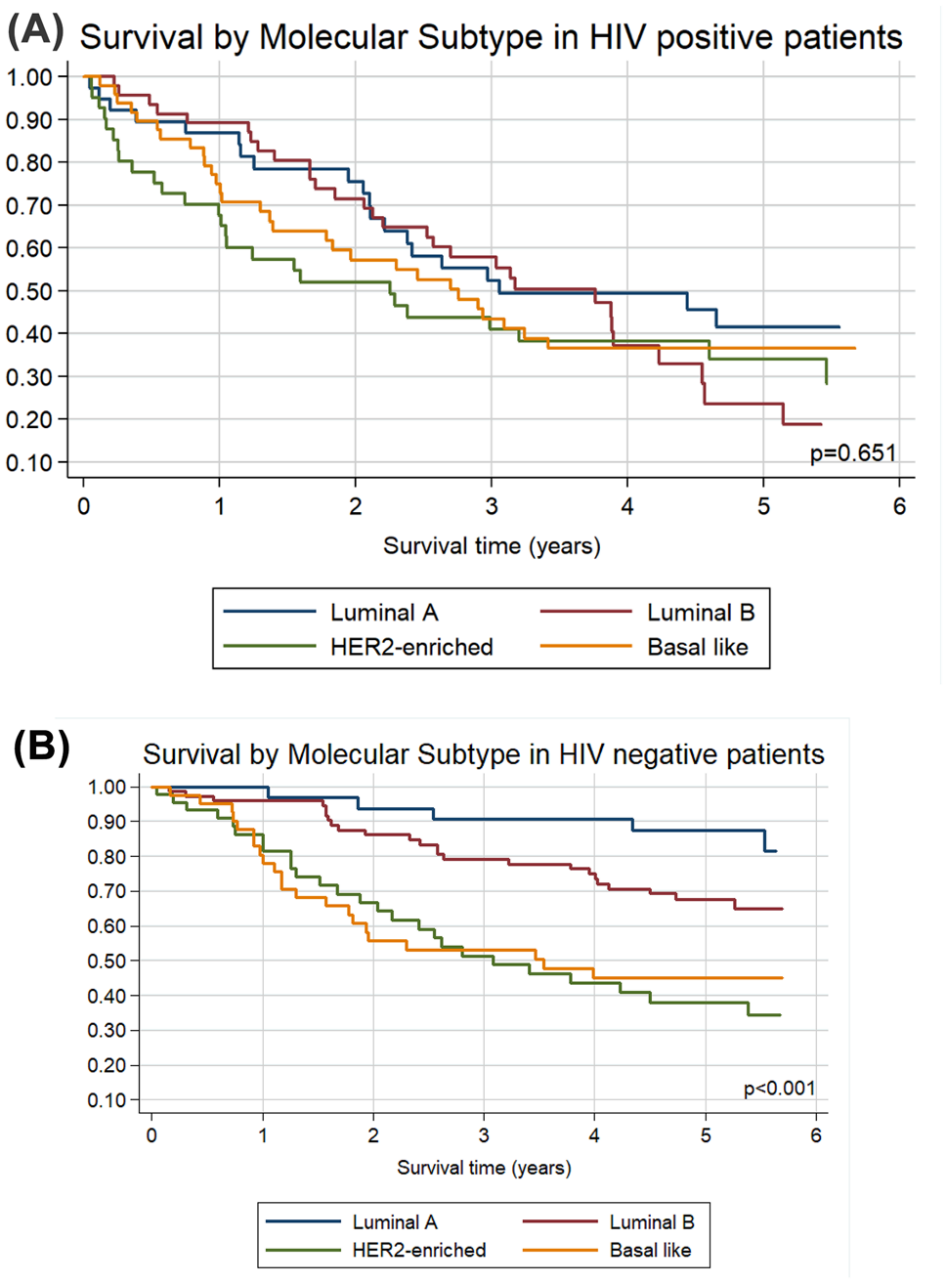
Overall survival by molecular subtype in (**A**) 175 HIV-positive patients; Unadjusted hazard ratios (95% CIs) compared to Luminal A: Luminal B 1.18 (0.68 – 2.07), *p* = 0.555; HER2-enriched 1.45 (0.81 – 2.58), *p* = 0.207; Basal-like 1.23 (0.73 – 2.17), *p* = 0.462. **B** 197 HIV-negative patients; Unadjusted hazard ratios (95% CIs) compared to Luminal A: Luminal B 2.39 (0.91 – 6.26), *p* = 0.077; HER2-enriched 6.09 (2.34 – 15.90), *p* < 0.001; Basal-like 5.47 (2.07 – 14.48), *p* = 0.001

**Fig. 3:**
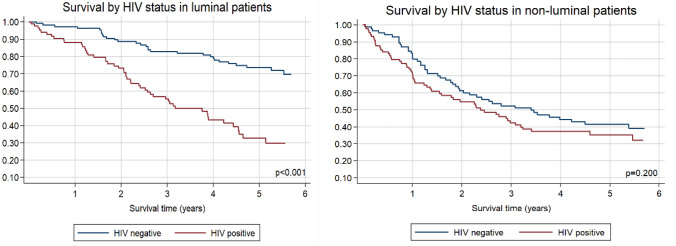
Overall survival by HIV status for (**A**) 195 patients with luminal breast cancer, unadjusted hazard ratio (95% CI) compared to HIV-negative: HIV-positive 3.63 (2.28—5.78), *p* < 0.001; and (**B**) 177 patients with non-luminal breast cancer, unadjusted hazard ratio (95% CI) compared to HIV-negative: HIV-positive 1.29 (0.87—1.90), *p* = 0.202

The study participants’ 5-year overall survival was 48.0%. Patients with the luminal A and luminal B intrinsic subtypes had better survival than those who had HER2-enriched or basal-like intrinsic subtypes (Fig. 1 and Table 2). As expected, the patients with the highest ROR scores had poorer survival than those with intermediate or low ROR scores (Table 2). Regardless of the intrinsic subtype, HIV-positive patients had poorer 5-year survival than HIV-negative patients (34.1% vs 59.6%, *p* < 0.001). Only among patients with luminal subtypes was HIV status associated with survival (Fig. 2 & 3).

**Table 2:** Unadjusted and adjusted breast cancer mortality hazard ratios

	Unadjusted Hazard Ratios	*p*-value	Adjusted Hazard Ratios*	*p*-value
Age at diagnosis				
< 50 years	1 (Ref)		1 (Ref)	
≥ 50 years	0.71 (0.52–0.95)	**0.024**	0.97 (0.71–1.32)	0.829
Linear Trend	0.99 (0.98–1.00)	0.131	1.01 (0.99–1.02)	0.483
Molecular Subtype				
Luminal A	1 (Ref)		1 (Ref)	
Luminal B	1.24 (0.78–1.98)	0.365	1.09 (0.68–1.75)	0.716
HER2-enriched	2.38 (1.49–3.80)	** < 0.001**	1.85 (1.15–2.98)	**0.011**
;Basal like	2.07 (1.29–3.33)	**0.003**	1.93 (1.20–3.10)	**0.007**
HIV status				
;HIV negative	1 (Ref)		1 (Ref)	
;HIV positive	2.08 (1.55–2.80)	** < 0.001**	2.14 (1.58–2.90)	** < 0.001**
Stage at diagnosis				
Early stage (1 and 2)	1 (Ref)		1 (Ref)	
Advanced stage (3 and 4)	3.59 (2.56–5.02)	** < 0.001**	3.65 (2.60–5.12)	** < 0.001**
Risk of recurrence				
Low	1 (Ref)		1 (Ref)	
Intermediate	0.78 (0.30–2.03)	0.615	1.00 (0.38–2.62)	0.993
High	2.66 (1.36–5.19)	**0.004**	2.18 (1.11–4.28)	**0.023**
Linear Trend	1.01 (1.01–1.02)	**0.002**	1.01 (1.00–1.02)	**0.049**

*Adjusted for age, stage, HIV status

Patients aged 50 years or more had better survival than younger patients in an unadjusted model (*p* = 0.024) but not in a model adjusted for stage and HIV status (*p* = 0.829) (Table 2). Among HIV-positive patients, the duration of HIV infection and ART use had no association with the overall survival.

### Disease-free survival

**Fig. 4:**
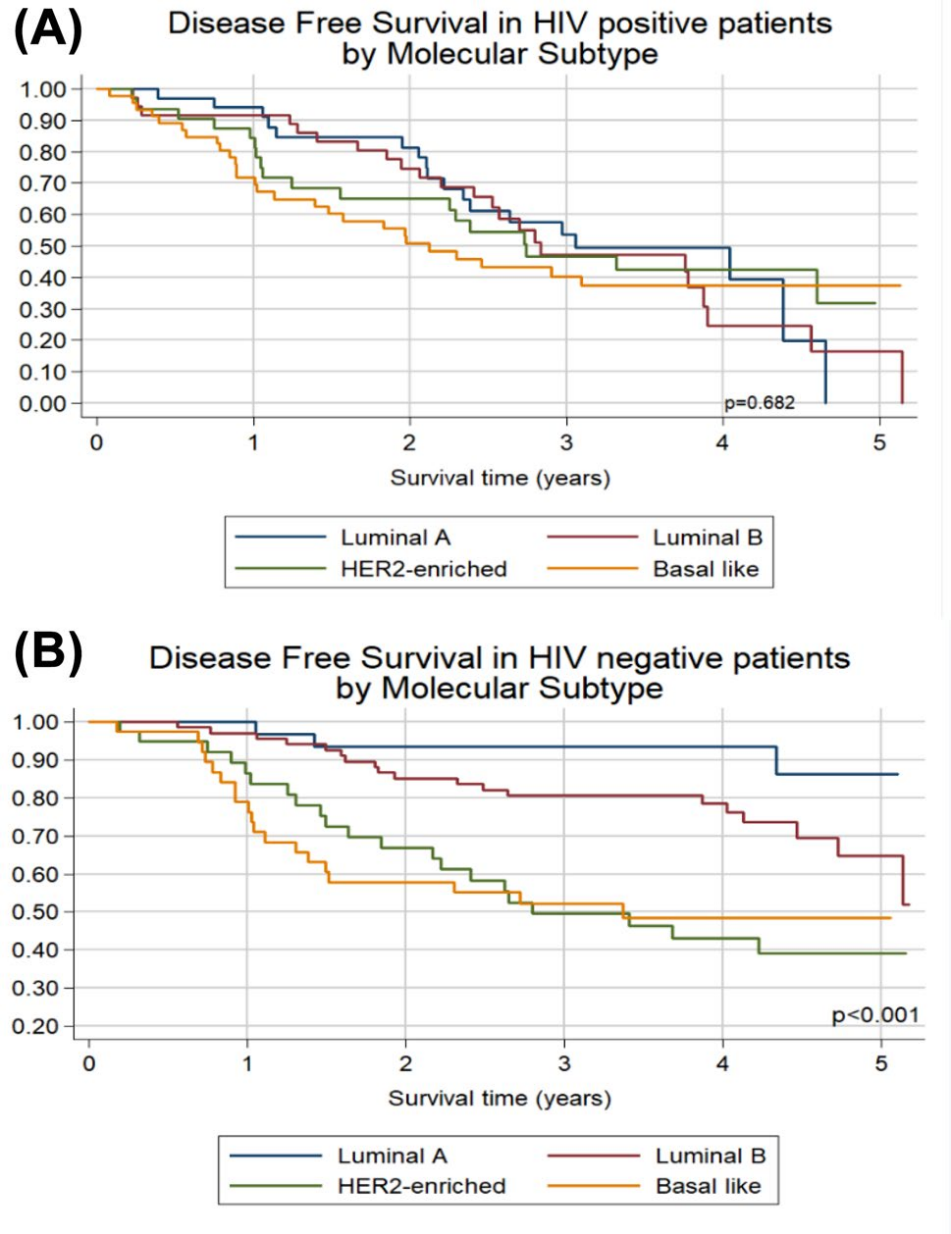
Disease-free survival by molecular subtype in (**A**) 151 HIV-positive patients; Unadjusted hazard ratios (95% CI) compared to Luminal A: Luminal B 1.18 (0.64 – 2.19), *p* = 0.601; HER2-enriched 1.06 (0.55 – 2.05), *p* = 0.864; Basal like 1.40 (0.77 – 2.55), *p* = 0.269. (B) 179 HIV-negative patients; Unadjusted hazard ratios (95% CI) compared to Luminal A: Luminal B 2.53 (0.86 – 7.42), *p* = 0.091; HER2-enriched 5.86 (2.00 – 17.12), *p* = 0.001; Basal like 6.46 (2.19 – 19.08), *p* = 0.001

Overall 5-year disease-free survival (DFS) was 45.4%, but it was far worse among HIV-positive than HIV-negative patients (23.9% vs 59.6%, *p* < 0.001). The HER-2 and basal-like intrinsic subtypes were associated with poorer DFS than Luminal A and luminal B intrinsic subtypes among HIV-negative patients but there was no statistical difference in DFS by molecular subtype in HIV-positive patients (Fig. 4). Patients with high ROR scores had very poor DFS (Table 3). Of 248 patients, 67 (27%) had breast cancer recurrence and their overall median (IQR) time to recurrence was 2 (1 – 3) years. The commonest site of disease recurrence was the lung, accounting for 45% of all sites. HER2-enriched and Basal-like intrinsic subtypes spread predominantly to the lungs while the Luminal B intrinsic subtype spread mainly to the liver. HIV infection had no impact on the site of distant metastasis.

**Table 3:** Unadjusted and adjusted hazard ratios for disease recurrence

Hazard Ratios for Disease Free Survival						
	Unadjusted	*p*-value		Adjusted for age, stage, hiv	*p*-value	
	Hazard Ratio			Hazard Ratio		
Age at diagnosis						
** < 50 years**	1 (Ref)			1 (Ref)		
≥ 50 years	0.63 (0.45–0.88)	0,007		0.86 (0.60–1.22)	0,397	
Linear Trend	0.99 (0.97–1.00)	0,134		1.00 (0.98–1.02)	0,904	
Molecular Subtype						
Luminal A	1 (Ref)			1 (Ref)		
** Luminal B**	1.24 (0.73–2.09)	0,429		1.19 (0.70–2.02)	0,521	
Her2-enriched	2.03 (1.20–3.45)	0,009		1.56 (0.91–2.68)	0,104	
;Basal like	2.37 (1.41–3.98)	**0,001**		2.29 (1.37–3.85)	**0,002**	
**HIV status**						
;HIV negative	1 (Ref)			1 (Ref)		
;HIV positive	2.30 (1.65–3.19)	** < 0.001**		2.26 (1.62–3.16)	** < 0.001**	
**Stage at diagnosis**						
Early stage (1 and 2)	1 (Ref)			1 (Ref)		
Advanced stage (3 and 4)	2.82 (2.00–3.97)	< 0.001		2.77 (1.96–3.90)	< 0.001	
Risk of recurrence						
Low	1 (Ref)		1 (Ref)			
Intermediate	0.84 (0.29–2.40)	0,739	1.11 (0.39–3.20)			0,844
High	2.88 (1.35–6.15)	0,006	2.48 (1.15–5.31)			0,02
Linear Trend	1.01 (1.00–1.02)	0,014	1.01 (1.66–3.24)			< 0.001
Viral Load						
Undetectable	1 (Ref)		1 (Ref)			
Detectable	0.86 (0.54–1.35)	0,503	0.73 (0.46–1.16)			0,178
ART status						
No	1 (Ref)		1 (Ref)			
Yes	0.83 (0.51–1.37)	0,466	0.89 (0.53–1.49)			0,657
Duration of ART						
≤ 1 year	1 (Ref)		1 (Ref)			
> 1 year	0.68 (0.36–1.29)	0,236	0.68 (0.36–1.29)			0,236
CD4 count						
< 200	1 (Ref)		1 (Ref)			
≥ 200	0.53 (0.30–0.95)	0,032	0.60 (0.33–1.07)			0,082

*Adjusted for age, stage, HIV status

## Discussion

Our findings of 48% 5-year overall survival and 45.4% disease-free survival are similar to those of other studies showing 50% 3-year survival in LMICs and 90% 5-year survival in HICs [2, 20]. Several features of our cohort may explain its poor survival. Overall in the SABCHO cohort, the median age at breast cancer diagnosis was 54 years, and HIV-positive patients were younger than HIV-negative patients (44 vs 57 years, < 0.001) [3, 7]. Moreover, the overall median age was younger than that in a cohort in the United States [21]. In the current sub-study, HIV- positive and HIV-negative patients were age matched, hence, our cohort’s median age was 48 years. In Norway, women younger than 50 years had a twofold risk for mortality compared to women 50- 59 years of age [22]. However, after adjusting for stage and HIV status, our study participants younger and older than 50 years did not differ in survival.

Breast cancer diagnosis at advanced stage is another known predictor of poor survival [6]. In our cohort, advanced disease at presentation was associated with poor overall and disease-free survival, and 56.6% of our patients had an advanced (Stage III/ IV) disease on presentation; similar to patients in a study in Tanzania (53.2%) [23], while in a study in Rwanda, more than 75% of patients presented with advanced disease [26]. In contrast, in the United States, less than 20% of patients had advanced breast cancer at the time of diagnosis [24]. Several patient-related and healthcare facility-related factors contribute to delayed presentation and late stage at diagnosis of breast cancer [25–28].

Although the direct effect of HIV infection on breast cancer progression is not yet fully understood, HIV infection among breast cancer patients has been associated with poor survival [3, 5, 6], except among patients with metastatic breast cancer [29]. In our cohort of patients diagnosed in stages I-III, HIV-positive status was associated with poor breast cancer survival.

Another known prognostic factor is the intrinsic breast cancer subtype. Triple-negative and HER2-enriched intrinsic tumours are more aggressive than luminal A and luminal B tumours and are associated with reported 2.5 and threefold risks of mortality [3, 7, 21, 30, 31]. About 20% of breast cancers are HER2-positive; the subtype associated with poorer clinico-pathological outcome features: younger age, larger size, lymph node involvement, increased nuclear grade, and negative hormone receptors [32, 33]. Moreover, it is associated with an increased risk for loco-regional and distant site recurrence, including a > 50% risk of developing central nervous system metastases [34]. About 15% of breast cancers are triple-negative, and that subtype is associated with young age at diagnosis, African descent, and BRCA 1 gene mutations [33]. It is also associated with poor disease-free and overall survival and with metastasis to the lungs and central nervous system [33–36]. In this cohort, the PAM50-based HER2-enriched and basal-like (triple negative) subtypes were associated with poor survival, even after adjusting for age, stage, and HIV status. Moreover, they accounted for 23.9% and 24.7%, respectively, of all the intrinsic subtypes in our cohort, higher prevalence than previously described (i.e., 20% and 15%, respectively) [32, 33, 37, 38]. In our cohort, HIV infection was not associated with PAM50 intrinsic subtype and these findings were also reported by several other studies, but a Mozambique-based study found that a higher proportion of HIV-positive than HIV-negative patients had triple-negative breast cancers [31, 39, 40]. Regarding survival, in our cohort HIV-negative patients with luminal breast cancer subtypes had significantly better survival than patients with non-luminal breast cancer subtypes. Interestingly, HIV-positive patients, did not differ in overall survival by molecular subtype. It is known that luminal breast cancer subtypes are less aggressive and are associated with more favourable outcomes than non-luminal breast cancer subtypes [3, 30, 33]. How HIV infection adversely affected the survival of patients with luminal breast cancer subtypes in our cohort still needs to be determined. Ayeni et al. recently reported an increased rate of non-compliance to prescribed tamoxifen treatment among HIV-positive patients with luminal breast cancer subtypes [41]. Tamoxifen is a selective estrogen receptor modulator proven to improve the survival of patients with luminal breast cancer subtypes [42].

In our study, the most common site for distant metastasis among patients with HER2-enriched or basal-like tumours was the lung, similar to what has been reported by other studies [33–36]. The liver and lung were the commonest sites of metastases for luminal B intrinsic subtypes, respectively, while the Luminal A subtypes were evenly spread between sites. HIV status had no impact on the site of distant metastases.

The ROR score is based on the measurement of the 50 genes included in the PAM50 assay, and the size of the tumour itself [9]. Low, intermediate, and high-risk groups by ROR have an estimated 10-year distant recurrence-free survival of: 96.7%, 91.3% and 79.9% respectively [9, 14]. In this study, 82.1% of the patients were in the high-risk category by ROR score and had relatively poor overall and disease-free survival. However, HIV status did not affect the ROR score.

The strength of this study is its duration of follow-up, which yielded 5-year overall and disease-free survival, matching the international standard, unlike most studies of breast cancer survival in LMICs, which have typically reported survival up to 4 years. Moreover, our study is the first, to our knowledge, to use the gene expression assay PAM50 to determine intrinsic subtypes and ROR among HIV-negative and HIV-positive patients. The limitations include small sample size and not being able to determine the breast cancer-specific mortality rate.

## Conclusion

In our cohort, HIV-negative status was associated with Luminal B intrinsic subtype. We also found that HIV infection, PAM50 HER2-enriched and basal-like intrinsic subtypes, and high ROR score were associated with poor overall and disease-free survival. Moreover, HIV-positive patients did not differ in the overall survival by molecular subtype, but HIV-negative patients with luminal breast cancer subtypes had better survival than those with other subtypes, (p < 0.001).

## Data Availability

Data is available and will be shared on request.
